# RNA-seq of macrophages of amoeboid or mesenchymal migratory phenotype due to specific structure of environment

**DOI:** 10.1038/sdata.2018.198

**Published:** 2018-10-02

**Authors:** Vladimír Čermák, Aneta Gandalovičová, Ladislav Merta, Jitka Fučíková, Radek Špíšek, Daniel Rösel, Jan Brábek

**Affiliations:** 1 Department of Cell Biology, Charles University, Viničná 7, Prague, Czech Republic; 2 Biotechnology and Biomedicine Centre of the Academy of Sciences and Charles University (BIOCEV), Průmyslová 595, 25242, Vestec u Prahy, Czech Republic; 3 Department of Immunology, Charles University, 2nd Faculty of Medicine and University hospital Motol, Prague, Czech Republic; 4 Sotio, Prague, Czech Republic

**Keywords:** Metastasis, Cell migration

## Abstract

M2-polarized macrophages have been shown to adapt their 3D migration mode to physical properties of surrounding extracellular matrix. They migrate in the integrin-mediated adhesion and proteolytic activity-dependent “mesenchymal” mode in stiff matrices and in the integrin and protease-independent “amoeboid” mode in low density, porous environments. To find out what impact the switching between the migration modes has on expression of both protein-coding and non-coding genes we employed RNA sequencing of total RNA depleted of ribosomal RNA isolated from macrophages migrating in either mode in 3D collagens. Differentially expressed genes from both categories have been detected and the changes in expression of selected genes were further validated with RT-qPCR. The acquired data will facilitate better understanding of how mechanical properties of tissue microenvironment reflect in macrophage immune function and how the transitions between mesenchymal and amoeboid migratory modes are regulated at the gene expression level.

## Background & Summary

Cell invasion is required for many physiological processes such as development, immune response or wound healing, and, in effect, many cell types possess the capability to invade^1^. Immune cells have developed very effective invasion strategies that enable them to perform immune surveillance even deep in tissues. However, cell invasion also facilitates dangerous pathological states such as cancer metastasis, and cancer cells are often considered to be masters of invasion. A key feature underlying effective invasion, shared by both immune cells and cancer cells, is the ability to adapt to different conditions by adjusting the mode of invasion, often referred to as invasion plasticity^2–5^.

The various invasion modes cells can adopt include two distinct individual invasion modes – the protease-dependent mesenchymal mode and protease-independent amoeboid mode. Generally, mesenchymal migration relies on the formation of adhesions to the extracellular matrix (ECM) and proteolytic degradation of adjacent fibres^6,7^. On the contrary, amoeboid cells do not rely on fibre degradation and instead dynamically deform their cell body (including the nucleus), to squeeze through pores in the ECM^8,9^. These invasion modes are interchangeable and cells can undergo the mesenchymal-amoeboid (MAT) or amoeboid-mesenchymal transitions (AMT) in response to intracellular and/or extracellular cues^6,10–14^. While processes of MAT and AMT in cancer cells have been extensively studied during the past years, little information is available on the molecular mechanisms behind the invasion plasticity of immune cells^3,15^.

An elaborate study by Cougole *et al.* tested the invasion plasticity of various immune cells in response to different ECM conditions and showed that these cells differ in their capability to adjust to soft or stiff matrices. They conclude that all leukocytes use the amoeboid mode, but only M2 macrophages possessed the ability to adjust their morphology according to the extracellular matrix conditions and switch between the amoeboid and mesenchymal phenotype^15^.

This phenomenon was described in detail by Van Goethem *et al.*, which showed that M2 macrophages modulate their invasion mode according to the ECM architecture^3^. In Matrigel and gelled collagen, both dense environments with small pores, M2 macrophages utilize the mesenchymal mode and form podosomes - membrane protrusions with proteolytic activity. Interestingly, this invasion mode can be inhibited by a protease inhibitor mix, but not by a selective inhibitor of matrix metalloproteinases alone, indicating other proteases to be involved. Moreover, protease inhibition does not result in MAT, as described in cancer cells. In fibrillar collagen, which permits cells to take advantage of larger pre-existing pores, macrophages are amoeboid and do not form podosomes. Amoeboid macrophage invasion is unaffected by protease inhibitors but is decreased in the presence of ROCK inhibitors, which is in line with observations of amoeboid cancer cells.

**Figure 1:**
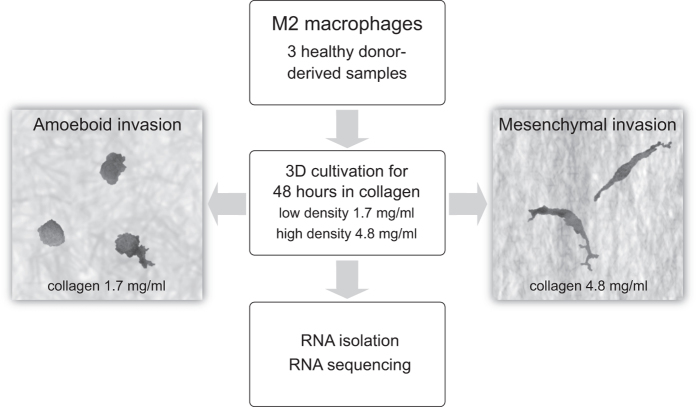
Schematic overview and experimental design of the study.

**Figure 2:**
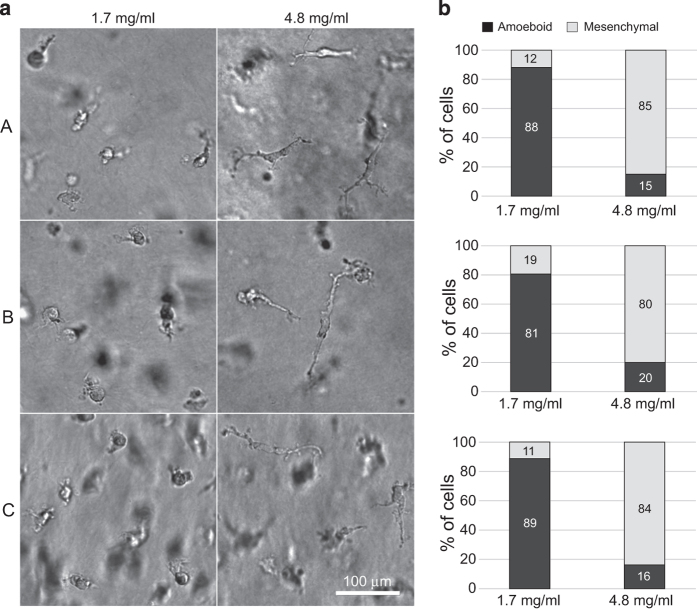
Morphology analysis of M2 macrophage samples embedded in low- and high-density collagen. (**a**) Representative images of the three analysed samples of M2 macrophages (A–C) embedded in 1.7 mg/ml or 4.8 mg/ml collagen. Images were acquired using Nikon-Eclipse TE2000-S, 10x objective with Hoffman modulation contrast. (**b**) Quantification of cell phenotype of the three analysed samples of M2 macrophages (**a**–**c**) embedded in 1.7 mg/ml or 4.8 mg/ml collagen.

To elucidate signalling driving this invasion adaptability of macrophages and potentially reveal candidate marker genes of the amoeboid and mesenchymal phenotype, we performed RNA sequencing of three independent primary samples of human M2-polarized macrophages embedded in both low and high-density collagen (Fig. 1), as described by Van Goethem *et al.*^3^ In agreement with their results, if we embedded M2 macrophages in 1.7 mg/ml collagen, they adopted the amoeboid phenotype in over 86% (SE 4.36%). In dense collagen (4.8 mg/ml), the majority (83%, SE 2.65%) of macrophages was mesenchymal (Fig. 2). To verify the migratory potential of both amoeboid and mesenchymal macrophage invasion modes, we performed time-lapse live cell imaging. Supplementary videos clearly demonstrate amoeboid locomotion in fibrillar collagen (Macrophage phenotype in fibrillar colagen, Data Citation 1) and document mesenchymal invasion of M2 macrophages in high-density environment (Macrophage phenotype in dense collagen, Data Citation 1).

A substantial advantage of primary M2 macrophages as a model system is their inherent invasion plasticity that enables to study mechanisms of MAT and AMT without any genetic manipulation or chemical treatment. The presented data will help to better comprehend the *in vivo* plasticity of cellular invasion caused by ECM diversity. In addition, analysis of the data revealed that the immune function of macrophages is also affected by mechanical properties of the ECM, hence reuse of the data by the immunological community could be beneficial.

## Methods

### Differentiation and M2 polarization of primary human macrophages

Primary human macrophages derived from CD14^+^ subpopulation of peripheral blood mononuclear cells from healthy donors aged between 25–40 years were differentiated and M2-polarized with M-CSF (100 ng/ml) in RPMI-1640 medium supplemented with 10% fetal bovine serum for 5–6 days before the experiments. Informed consent was obtained from all subjects. The experiments were approved by the Ethical Committee of the Second Faculty of Medicine at Charles University in Prague.

### Three-dimensional cell culture and RNA isolation

Five million M2 macrophages were cultured in 500 μl of three-dimensional gel made of bovine skin collagen (Nutragen, Advanced BioMatrix, 1.7 or 4.8 mg/ml) in RPMI-1640 with 1% fetal bovine serum and 100 ng/ml M-CSF (Peprotech) for 48 hours in a 24-well plate. Gels from two wells were transferred into one 2 ml tube and homogenized with Tissue Tearor (BioSpec Products) in 600 μl of RNA extraction solution (60% v/v water-saturated phenol, 3.25 M guanidine thiocyanate, 400 mM sodium acetate buffer pH 4.0, 0.4% w/v N-lauroylsarcosine, 160 mM 2-mercaptoethanol) plus 100 μl of 6.1 M sodium chloride.

200 μl of chlorophorm was added to approx. 1 ml of the lysate and the mixture was vortexed vigorously for 10 seconds. After 30-minute centrifugation at 18,000 g at 4 °C, the polar upper phase was transferred into a new tube, volume adjusted to 800 μl with RNase-free water, RNA was precipitated with 600 μl of isopropanol at −20 °C overnight and recovered by centrifugation at 18,000 g for 30 minutes at 4 °C. The RNA pellet was washed three times with 600 μl of 75% ethanol and air-dried. Next, the RNA was treated with DNase I to remove possible genomic DNA contamination. To this end, the pellet was directly dissolved in 100 μl of a solution containing 4 units of DNase I (Thermo Fisher Scientific) and manufacturer-provided reaction buffer, and incubated at 37 °C for 30 minutes. After that, the RNA was re-purified with RNeasy Protect Mini Kit (Qiagen) according to the manufacturer’s instructions and eluted in 50 μl of RNase-free water (Invitrogen).

### Analysis of cell morphology in 3D collagen

Cell morphology was analysed with ImageJ software by measuring longest axis to shortest axis ratios. Cells with the ratio equal to 2.5 or larger were considered mesenchymal, as determined earlier by Van Goethem *et al.*^3^. For each condition a minimum of 100 cells was analysed.

### RNA sequencing and data processing

Stranded, Illumina HiSeq-compatible library was constructed with ScriptSeq Complete (Human/Mouse/Rat) library preparation kit (Epicentre) according to the manufacturer’s instructions. An equimolar pool of 6 sample libraries (three donor-matched pairs) was sequenced on one whole lane of Illumina HiSeq 2500 sequencer in high output, paired (2 × 125 cycles) mode by Beckman Coulter Genomics.

Raw reads were trimmed of adapter sequences with Cutadapt^16^ (version 1.15) and mapped to human genome version GRCh38.91 with the STAR short read aligner^17^ version 2.5.4b with default settings and output extended with read counts per gene.

Adapter-trimmed reads were deposited in the ArrayExpress database (Data Citation 2). The samples and related files are summarized in Table 1.

### Differential Gene Expression Analysis

**Figure 3:**
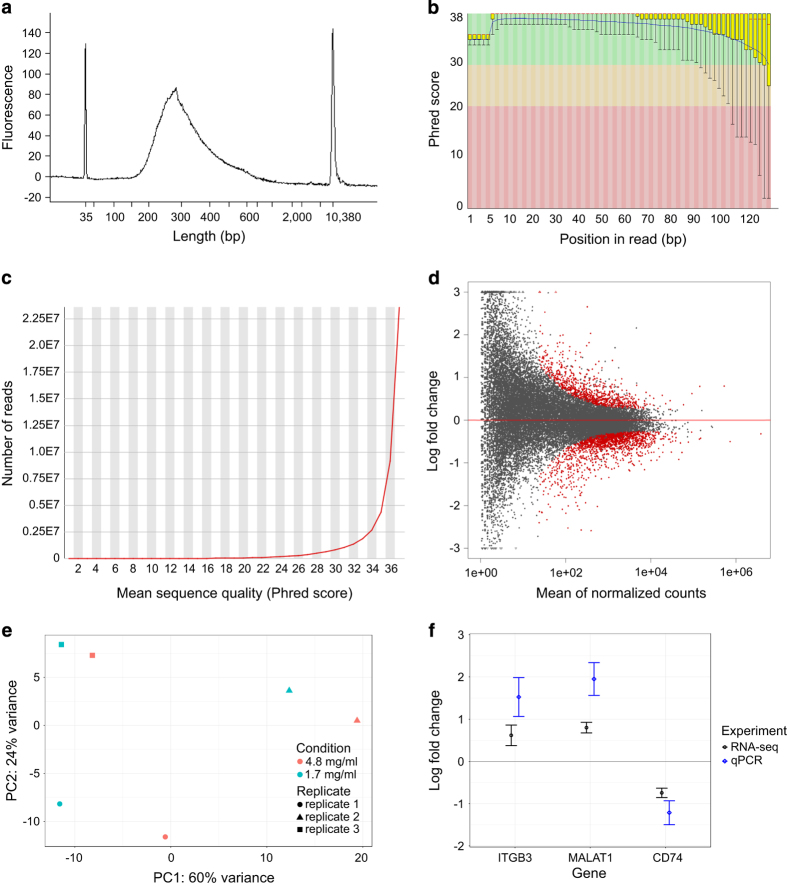
RNA-seq results validation. (**a**) Sequencing library fragment size distribution of sample 1. (**b**) Per base sequence quality expressed as Phred score by position, sample 1, first reads. (**c**) Quality score distribution over all reads of sample 1. (**d**) MA plot of log2 fold change values against normalized counts for each gene in the analysis. Red points mark genes with FDR < 0.1. (**e**) Principal component analysis of gene expression profiles. (**f**) Comparison of log2 fold change values detected by RNA-seq and RT-qPCR, respectively for the indicated genes.

To find differentially expressed genes R package DESeq2^18^ (version 1.18.1) was used with the raw read count output of the STAR aligner as the input. Default workflow with a design formula reflecting paired nature of the samples was applied. Differentially expressed genes with FDR < 0.1 (874 upregulated and 1,676 downregulated genes) are available in the file Differentially_expressed_genes.xlsx (Data Citation 1) and their distribution by gene expression level is depicted as MA plot in Fig. 3d. Principle component analysis of the gene expression profiles showed dominant clustering of the samples by individual donors (Fig. 3e). This obviously reflects the well-known high variation of primary cell samples that can much exceed the variation brought about by experimental treatment as in this case. The use of two-factor design (cell donor and collagen concentration) in the statistical analysis is thus crucial.

### Analysis of ribosomal cDNA content and strandedness of libraries

Adapter-trimmed reads were mapped to human ribosomal RNA coding sequences (NR_023379, NR_003285, NR_003286, NR_003287, NR_137294, and NR_137295) with BBMap aligner^19^ in local alignment mode. Ribosomal cDNA content in the libraries was expressed as percentage of reads mapped to nuclear or mitochondrial rRNA transcripts (the larger number from the paired mates). Strandedness of libraries was assessed from ratios of read counts mapped (by STAR) to annotated genomic loci in sense and antisense orientation. To eliminate the noise associated with low-expressed genes we limited the calculations to genes with lognormal mean or higher expression (approx. 6,000 genes). We also excluded genes with more antisense transcripts than those in sense orientation. The mean sense/antisense ratio was calculated as a geometric mean of all included gene ratios across all samples.

### Reverse Transcription – Quantitative polymerase chain reaction (RT-qPCR)

All the RT-qPCR experiments were performed according to MIQE guidelines^20^. Briefly, total RNA was extracted from cells embedded in 3D collagen matrix as described above. RNA reverse transcription was performed using M-MuLV Reverse Transcriptase (NEB) with 1 μM oligo-dT and 0.2 μM MALAT1-specific primer and 700 ng of total RNA. RT-qPCR was performed using SYBR green mix (1x Standard Taq buffer (NEB) with 3.0 mM MgCl_2_, 2 U/100 μl Hot Start Taq DNA Polymerase (NEB), 10% glycerol (Carl Roth), 6% DMSO (Sigma), 200 μM dNTPs (Thermo Fischer Scientific), 0.05% Triton X-100 (Sigma), 1x SYBR® Green I Nucleic Acid Stain (Lonza), and Ultrapure Water (Invitrogen)) and CFX384 Real Time PCR Instrument (Bio-Rad). Annealing and elongation temperatures were 63 °C and 68 °C, respectively. The RT-qPCR reaction volume was 10 μl with 0.5 μl of cDNA and 200 nM primers (for primer details see Table 2). Samples were run as technical quadruplicates using FrameStar® 480/384 with RT-qPCR Adhesive seal (4titude) 384-well plates. Cq and relative expression values were calculated by setting single threshold value for each target and further analysed by qBase + software (Biogazelle)^21^ using EIF4H, HNRNPL and PPIA as reference gene index (based on geNORM analysis^22^). Amplification efficiencies were determined by the standard curve analysis. Log2 fold change values were calculated from relative expression values.

## Data Records

Time-lapse movies documenting migratory phenotypes of the cells and a table listing all the differentially expressed genes detected in the study were deposited in the Figshare repository (Data Citation 1).

RNA-seq data have been deposited in the ArrayExpress database at EMBL-EBI (Data Citation 2).

## Technical Validation

Viability and morphological phenotype of the cells was verified by wide field microscopy (Fig. 2). The integrity of isolated total RNA was confirmed with agarose gel electrophoresis of denatured RNA samples. The quality and size distribution of sequencing libraries was analysed with Agilent BioAnalyzer 2100 (Fig. 3a). The profiles, according to expectation, showed peak position around 280 bp and a longer right tail. The output statistics of STAR aligner showed an average paired read length of 230 bases. Raw reads trimmed of adapter sequences, approx. 2 × 40 million reads per sample, were quality-checked with FastQC software^23^. FastQC’s plots of Phred scores by position (Fig. 3b) showed typical profiles with decreasing quality towards the ends of reads. The overall quality was analysed with BBTools’ Reformat utility^19^ detecting approx. 91% bases having Phred score 30 or better. The distribution of quality scores is presented in Fig. 3c. Figure 3 displays quality assessment results for sample 1. Complete results for all six samples are provided in Supplementary Figure 1. FastQC analysis also detected a significant presence of duplicate reads, which was expected as the library construction involved a PCR amplification step. The STAR aligner uniquely mapped approx. 90% of the fragments to human genome, approx. 6% fragments were multi-mapped, and approx. 4% fragments were excluded as too short. We further analysed residual rRNA content after ribodepletion expressed as proportion of reads mapped to rRNA sequences. Only 0.3–1.1% reads could be mapped to nuclear encoded rRNA. However, 9.5–16% of all reads were mapped to mitochondrial 12S and 16S rRNA sequences with higher mtrRNA content in all high-concentration collagen samples (Table 3). The Ribo-Zero component of the library construction kit used in this work does not deplete total RNA of mitochondrial rRNA, so this is not unexpected.

The libraries were prepared as strand-specific. To analyse the level of strandedness in the sequencing data we compared proportions of sense and antisense reads mapped to known genes of intermediate to high expression. The average sense/antisense ratio was 18.4 ± 1.87 (geometric mean and standard deviation). This might appear slightly less than expected, however a few factors lowering apparent strandedness should be taken into account. The ribodepleted total RNA is much more complex than mRNA isolated with oligo-dT probes. It contains unprocessed primary transcripts that may partly span neighbouring genes in opposite orientation. It also contains large proportion of non-coding sequences that sometimes arise from antisense transcription of coding genes. Another source of partial loss of strandedness is the DNA-dependent DNA polymerase activity of reverse transcriptase.

To validate the results of differential gene expression analysis, RT-qPCR was performed with the RNA samples used for sequencing to compare changes in expression of three selected genes – ITGB3, MALAT1 and CD74 as detected by RNA-seq and RT-qPCR. These genes were chosen in order to represent different functional categories (cell adhesion, antigen presentation) and also both protein-coding and non-coding (MALAT1) genes. The results showed essentially the same expression pattern with RT-qPCR detecting greater fold-changes than RNA-seq (Fig. 3f).

## Additional information

**How to cite this article**: Čermák, V. *et al*. RNA-seq of macrophages of amoeboid or mesenchymal migratory phenotype due to specific structure of environment. *Sci. Data*. 5:180198 doi: 10.1038/sdata.2018.198 (2018).

**Publisher’s note**: Springer Nature remains neutral with regard to jurisdictional claims in published maps and institutional affiliations.

## Supplementary Material



Supplementary Information

Supplementary Table 1

## Figures and Tables

**Table 1:** Summary of sequencing data.

**Subjects**	**Gender**	**Condition**	**Protocols**	**ENA**	**BioSD**
Donor A	male	1.7 mg/ml collagen	P-MTAB-73510 to P-MTAB-73516	ERS2359742	SAMEA1054135
Donor A	male	4.8 mg/ml collagen	P-MTAB-73510 to P-MTAB-73516	ERS2359739	SAMEA1054132
Donor B	female	1.7 mg/ml collagen	P-MTAB-73510 to P-MTAB-73516	ERS2359743	SAMEA1054136
Donor B	female	4.8 mg/ml collagen	P-MTAB-73510 to P-MTAB-73516	ERS2359740	SAMEA1054133
Donor C	male	1.7 mg/ml collagen	P-MTAB-73510 to P-MTAB-73516	ERS2359744	SAMEA1054137
Donor C	male	4.8 mg/ml collagen	P-MTAB-73510 to P-MTAB-73516	ERS2359741	SAMEA1054134

**Table 2:** Primers used for RT-qPCR.

**Transcript**	**Acc. Number**	**Primer**	**Sequence**	**Product (bp)**	**Eff. (%)**
**MALAT1**	**NR_002819**	**MALAT1_5s**	GAATTGCGTCATTTAAAGCCTA	85	92.2
		**MALAT1_5a**	GTTTCATCCTACCACTCCCAAT		
**ITGB3**	**NM_002211**	**ITGB3f**	CACCATCCACGACCGAAAAG	82	99.5
		**ITGB3r**	CAGTGGGTTGTTGGCTGTGT		
**CD74**	**NM_001025159**	**CD74f**	GGAAGGTCTTTGAGAGCTGGAT	114	111.0
		**CD74r**	CTTCCTGGCACTTGGTCAGTA		
**HNRNPL**	**NM_001533**	**HNRNPLs**	CTGGAGGTGACCGAGGAGAA	96	99.3
		**HNRNPLa**	GCGCTCACTTTTGCCTGAGAA		
**EIF4H**	**NM_022170**	**EIF4Hs**	CTTCGACACCTACGACGATCG	95	97.7
		**EIF4Ha**	CCTTCTGGCTACGGGAACCAT		
**PPIA**	**NM_021130**	**PPIAf**	GCCGAGGAAAACCGTGTACTA	106	94.1
		**PPIAr**	CTGCAAACAGCTCAAAGGAGAC		

**Table 3:** Percentage of rRNA matching reads in RNA-seq data.

**Sample**		**Nuclear-encoded rRNA**	**Mitochondrial rRNA**
Donor A	1.7 mg/ml collagen	0.9%	9.5%
	4.8 mg/ml collagen	0.7%	11.8%
Donor B	1.7 mg/ml collagen	0.3%	9.8%
	4.8 mg/ml collagen	0.4%	16.0%
Donor C	1.7 mg/ml collagen	1.1%	11.0%
	4.8 mg/ml collagen	0.6%	13.5%
