# Supplementary figures and images for: RNA-seq of macrophages of amoeboid or mesenchymal migratory phenotype due to specific structure of environment
Source: Sci Data. 2018 Oct 2;5:180198. doi: 10.1038/sdata.2018.198 (PMC6167950; doi:10.1038/sdata.2018.198)

## Library profile

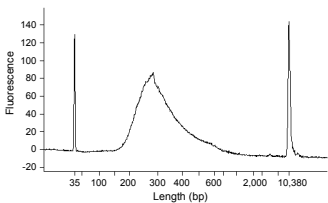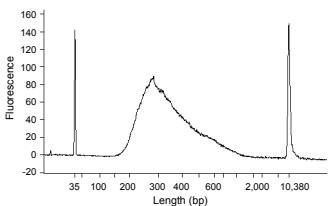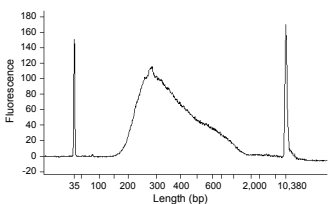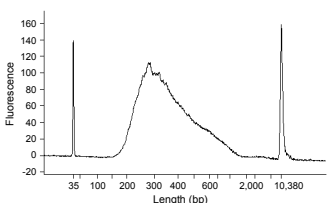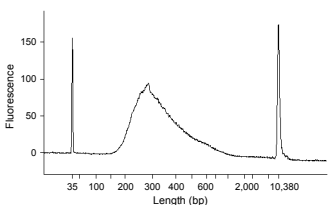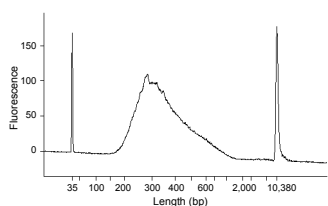

## Quality scores across all bases

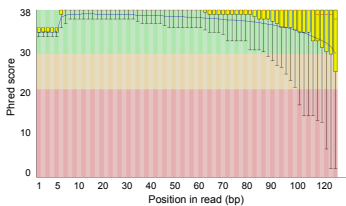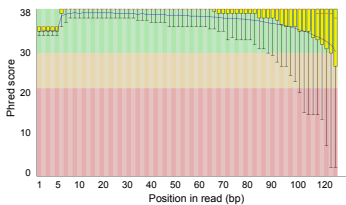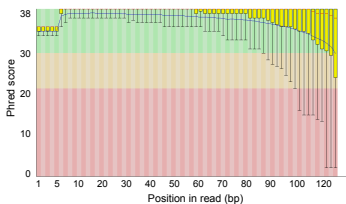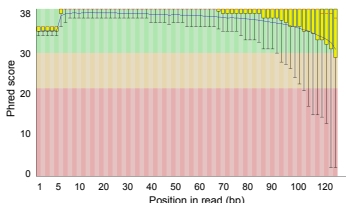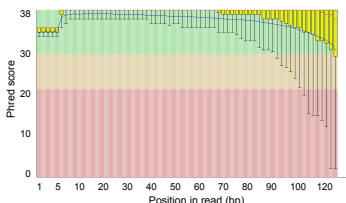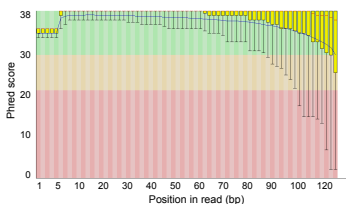

## Quality score distribution

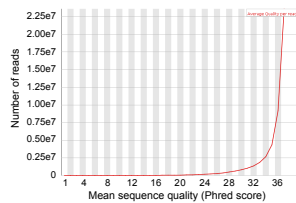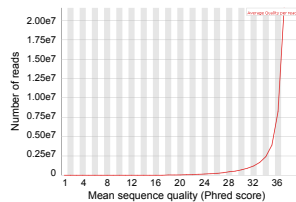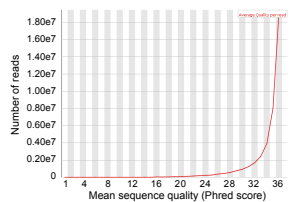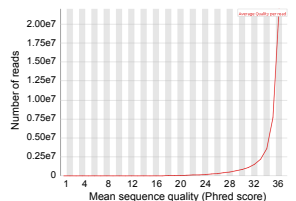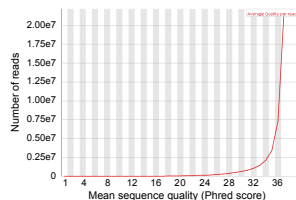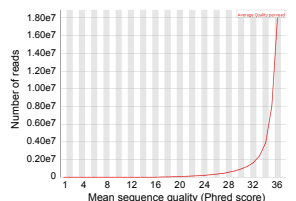

Supplement: Supplementary Information [file sdata2018198-s2.pdf]
